# The First Case of Familiar Anti-leucine-rich Glioma-Inactivated1 Autoimmune Encephalitis: A Case Report and Literature Review

**DOI:** 10.3389/fneur.2022.855383

**Published:** 2022-04-14

**Authors:** Chuhan Ding, Qibing Sun, Ran Li, Hanli Li, Yu Wang

**Affiliations:** Department of Neurology, The First Affiliated Hospital of Anhui Medical University, Hefei, China

**Keywords:** Anti-leucine-rich glioma-inactivated1 autoimmune encephalitis, autoimmune encephalitis, LGI1, HLA, case report

## Abstract

Anti-leucine-rich glioma-inactivated1 (Anti-LGI1) autoimmune encephalitis is a rare autoimmune disease discovered in recent years. It is generally not defined as an inherited disease, though its etiology is still unclear. Herein, we report the first case of adult patients with familial anti-LGI1 encephalitis. Two biological siblings who worked in different regions were successively diagnosed with anti-LGI1 encephalitis in their middle age. The two patients had similar clinical manifestations including imaging results. Their clinical symptoms improved after immunotherapy and antiepileptic therapy. Given that some unique human leukocyte antigen (HLA) subtypes appear at a high frequency, multiple recent studies have revealed that anti-LGI1 encephalitis is associated with genetic susceptibility. One of the patients underwent HLA genotyping and whole-exome sequencing (WES), revealing the same HLA typing as in previous studies and two rare HLA variants. Therefore, further studies involving larger samples and more populations should be conducted to explore the possibility of other influencing factors such as environmental impacts.

## Introduction

Anti-LGI1 encephalitis, a rare autoimmune encephalitis defined in recent years, is characterized by seizures, cognitive impairment, psychiatric disorders, and refractory hyponatremia. LGI1 is a type of neuron-secreted protein which dominantly expresses in the hippocampus and temporal cortex and transmits signals from the presynaptic to the posterior membrane (1, 2). Autoimmune encephalitis is a clinical syndrome of which the diagnosis is based on the detection of accurate antibodies, though it is not fully recognized. Currently, the first-line therapy for autoimmune encephalitis includes immunoglobulin, glucocorticoid, and plasma exchange, and the second-line therapy includes rituximab, cyclophosphamide, and mycophenolate mofetil (3). This report describes the first pair of siblings who were diagnosed with anti-LGI1 encephalitis after experiencing a convulsive seizure. According to previous gene association studies (3), we conducted genetic tests on the younger brother and found that his unique HLA haplotype was consistent with these studies, in addition to identifying two HLA variants. Therefore, more comprehensive genetic studies in a larger population are warranted.

## Patient 1

A 39-year-old man was admitted to our department in July 2021 because of one-month history of short-term memory loss and a generalized tonic-clonic seizure (GTCS) attacking during sleep. The patient had no personal history of hypertension, diabetes, or other diseases and had no alcohol consumption but smoking for 10 years.

Neurological examination revealed normal except the spatial and temporal disorientation and memory impairment, especially the short-term memory impairment. The patient was able to recall three items immediately but, afterward, unable to recall any one. His Mini-mental State Examination (MMSE) was scored 19/30, and Montreal Cognitive Assessment (MoCA) was scored 12/30.

In detail, he had mild impairment in naming, severe destruction in visuospatial abilities, executive functioning, sentence repetition, abstract thinking, orientation, and delayed recall.

**Figure 1:**
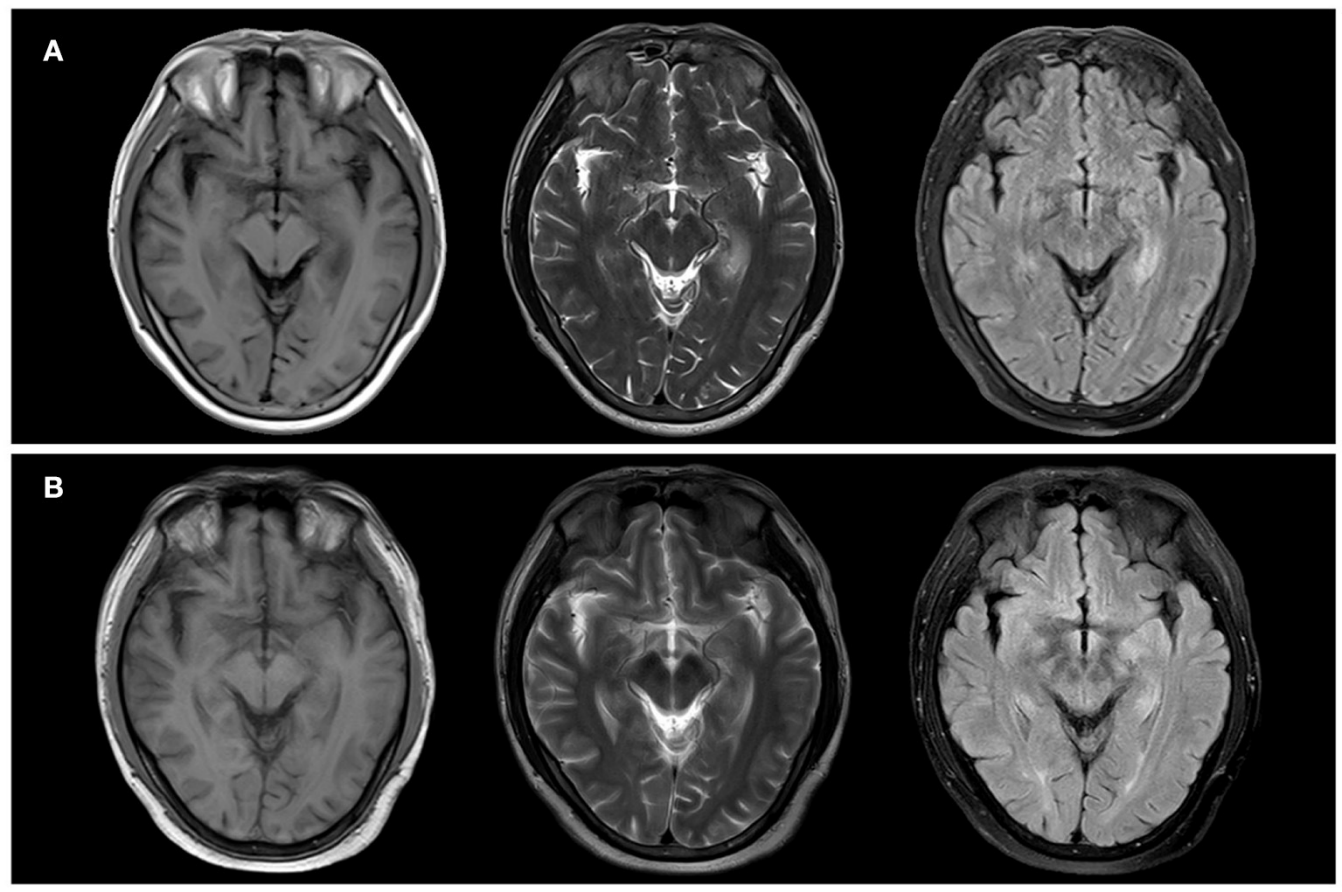
Neuroradiologic MRI (1.5 T) of Patient 1. The bilateral temporal lobes and bilateral hippocampus showed hypointensity on T1WI and hyperintensity on T2WI and FLAIR. Notably, they were more pronounced in the left side **(A)**. After two months, the bilateral temporal lobes and bilateral hippocampus showed a slightly lower signal on T1WI and a slightly higher signal on T2 and FLAIR. Notably, they were more pronounced in the left side **(B)**.

The brain magnetic resonance image (MRI) conducted on the admission day presented hyperintensities on T2 weighted image (T2WI) and fluid-attenuated inversion recovery (FLAIR) image but hypointensities on T1 weighted image (T1WI) in the bilateral temporal lobes and hippocampus with dominance in the left side (Figure 1A). Thoracic computed tomography (CT) revealed normal. The serum sodium concentration was 132.4 mmol/L which was lower than the normal (reference range: 137.0–147.0 mmol/L). The white blood cell count and C-reactive protein (CRP) were slightly higher than normal. The electroencephalogram (EEG) was detected with generalized slow wave (delta) activities. Cerebrospinal fluid (CSF) examination showed a slightly increased in the count of leukocytosis (7 × 10^6^/L) but normal in the levels of chloride, glucose, and protein. Autoimmune encephalitis antibodies were detected with positive LGI1 antibody in CSF (1:100+) and serum (1:1000+). The clinical information of the patient is indicated in Table 1.

**Table 1:** Clinical profiles of the two patients.

	**Test**	**Patient 1**	**Patient 2**
Characteristics	Gender	male	female
	Age at onset	39	36
Clinical Symptom	Central nervous system	Seizure, memory decline	Seizure, memory decline, disorder of behavior, hallucinations, blurred vision
	Seizure types	Generalized tonic-clonic seizures	Simple partial seizures
	Peripheral nervous system	no	Neuropathic pain, muscle weakness and numbness
	Autonomic nervous system	tachycardia	Hyperhidrosis
Laboratory Studies	CBC	WBC 10.5 × 10^9/^L, NUET 7.37 × 10^9^/L	WBC 9.80 × 10^9^/L
	Serum sodium	133.2 mmol/L	129 mmol/L
	Neoplasm	No	No effective basis for neoplasm
	Serological tumor markers	WNL	CA7-24: 8.72 U/mL
	Liver and kidney function tests	WNL	WNL
Brain Imaging	EEG	a delta (2-3c/s)activity	Extensive diffuse slow waves
	Initial MRI	Hyperintensities in bilateral parahippocampus	Hyperintensities in left temporal lobe
	Follow-up MRI	Slightly hiper signals on bilateral parahippocampus	None
LGI1-IgG	Serum (Cell-based assays, diluted 1:10)	1:1000+	1:100+
	CSF (Cell-based assays, without diluted)	1:100+	1:10+
Cerebrospinal Fluid Studies	Pressure	110 mm H_2_O	82 mm H_2_O
	Nucleated Cell Count	7 × 10^6^/L	1 × 10^6^/L
	Glucose	Normal	5.00 mmol/L
	Chloride	Normal	Normal
	Protein	Normal	Normal
	Microbiological and virological test	Normal	Normal
Other Auxiliary Examination	Chest CT	Normal	Normal
	Echocardiography	Sinus tachycardia	Normal
Treatment		Immunotherapy (IVIG and corticosteroids)	Immunotherapy (corticosteroids)
outcome		Returned to work	Returned to work

The patient was treated with intravenous immunoglobulin (IVIG) at a dose of 0.4 g/kg/day (25 g in total) for five days, followed by methylprednisolone pulse therapy (500 mg/day for five days, 250 mg/day for three days, 120 mg/day for three days). After treatment, his cognition improvement was remarkable from daily performance as he could realize he was a patient instead of mistaking himself as a caregiver for his wife, though no significant improvement in MMSE or MoCA scores after hospitalized treatment. He was discharged with oral prednisone and sustained-release sodium valproate tablets for seizure control. In a follow-up of three months after treatment, brain MRI imaging showed that the brain lesion improved (Figure 1B). The anti-LGI1 titer in serum decreased to 1:100+. MoCA score ameliorated to 15/30 for the noticeable improvement observed in visuospatial abilities, executive functioning, and retelling abilities. Nevertheless, severe impairment of orientation persisted.

## Patient 2

In 2017, a 36-year-old woman, the biological sister of patient 1, was admitted to another hospital due to paroxysmal full-body numbness, “paroxysmal twitch” in upper limbs, blurred vision, autonomic dysfunction, memory loss, confusion, and visual hallucinations. Notably, her clinical presentation first appeared two months before admission. However, after consulting in several hospitals, she was misdiagnosed with dysautonomia or peripheral neuropathy.

The medical record indicated she had depressed mood, poor orientation, and poor memory. The physical examination revealed mild weakness of extremities (muscle strength grade 5-/5), walking slowness, and unsteady gait.

**Figure 2:**
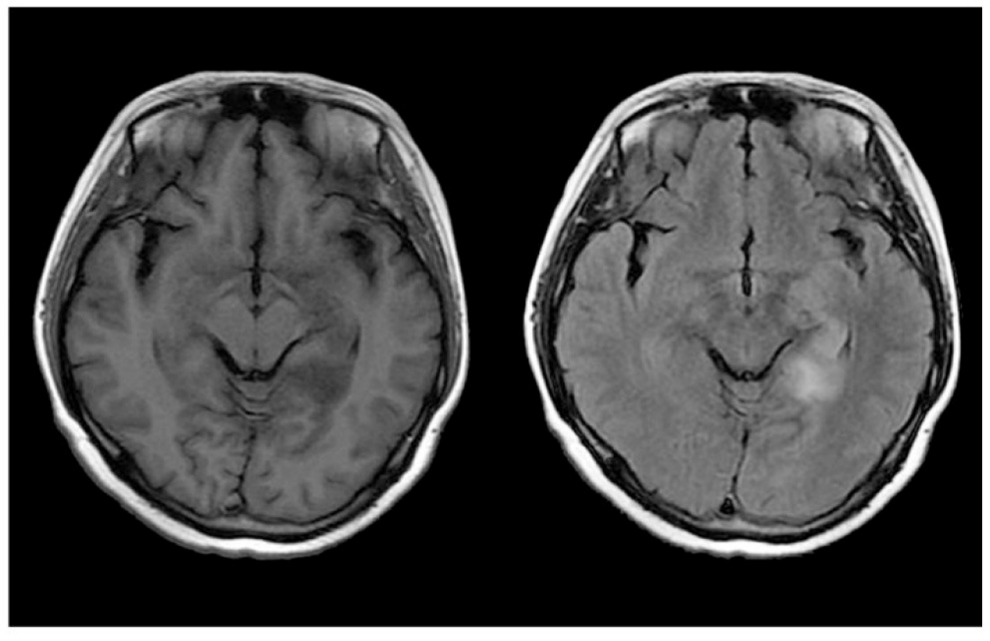
Neuroradiologic MRI (3.0 T) of Patient 2. The left temporal lobe and hippocampus showed hypointensity on T1WI and hyperintensity on FLAIR.

Her blood routine test, blood biochemistry test, and anti-nuclear antibodies were all within physiological ranges. With regard to tumor markers, carbohydrate antigen 724 was mildly increased to 8.72 U/mL (reference range: 0–8.20 U/mL), but no definite tumor was detected. Brain MRI plain scan and enhancement revealed anomalous signals in the left temporal lobe and hippocampus (Figure 2). In addition, EEG showed generalized extensive diffuse slow waves.

CSF examination indicated WBC count and protein level were normal, chloride slightly increased and glucose slightly increased. Autoimmune encephalitis antibodies test ultimately indicated that anti-LGI1 was positive in CSF (1:10+) and serum (1:100+). She was forthwith treated with sodium valproate tablets and methylprednisolone pulse therapy for 20 days with dosage decreasing and discharged with oral prednisolone. She had difficulties in follow-up due to busy workloads after discharge. Long-term administration of prednisolone made her look swollen, thus she eventually stopped taking medication after three years' oral hormone. She is still plagued by memory impairment while we followed her up, but she refused further treatment, examination, and further follow-up visits. This made it difficult to get her WES and other laboratory examination. She remained mild cognitive impairment with no disturbance of daily life as informed in a recent phone follow-up.

## Discussion

To date, almost all reported cases of autoimmune encephalitis were sporadic cases except for one familial autoimmune encephalitis reported in two pediatric brothers affected with voltage-gated potassium channel (VGKC) encephalitis (4).

Here, we report the first case of familiar LGI1 autoimmune encephalitis in adult patients. The clinical profiles are shown in Table 1. Specifically, two adult siblings at similar age were successively diagnosed with anti-LGI1 encephalitis within four years. Although both patients presented with subacute onset, and developed seizures and memory decline, some differences existed between the two siblings in brain images and EEG presentations as well as in clinical manifestations. The sister exhibited more severe and earlier autonomic dysfunction which had misled the physicians to diagnose it as peripheral neuropathy or autonomic dysfunction. Tests for autoimmune antibodies were undergone after her seizure attack, and this made the diagnosis of anti-LGI1 encephalitis established.

Recently, it was reported that adolescent siblings with acquired autoimmune syndrome after mercury exposure, and several autoantibodies including anti-LGI1 were detected (5). However, these two siblings we reported excluded toxicant exposure. As they had lived in distinct environments for more than 20 years before symptom onset, the living environment might not be a critically potential pathogenic factor.

Due to the consanguinity between the two patients, it should be taken into consideration that anti-LGI1 encephalitis is associated with genetic susceptibility. Multiple recent studies have indicated an association of anti-LGI1 encephalitis with HLA (6–11), but familial cases supporting this genetic association had never been reported. On the other hand, this definite and consistent genetic susceptibility has not been found in other types of autoimmune encephalitis. HLA genes are closely linked and obey Mendelian law of inheritance. Therefore, there is a 25% chance of two siblings being identical in the HLA genotype, a 50% chance of sharing the same HLA haplotype, and a 25% chance of not having the same HLA haplotype. It is well-known that HLA genes encode antigen-presenting proteins on the cell surface participating in the immune response directly. Studies have revealed genetic associations exist between HLA and various autoimmune diseases except for autoimmune encephalitis, among which HLA class II genes exert their effectiveness through autoantibody production. For instance, HLA haplotype DR3-DQ2 and DR4-DQ8 increase the risk of celiac disease (12), HLA-DR is associated with systemic lupus erythematosus and lupus nephritis (13), and HLA-DRB1^*^10:01-DQB1^*^05:01 is associated with IgLON5 encephalopathy (14). HLA class I genes are mostly expressed in diseases that do not produce antibodies, with the most famous example of the strong correlation between ankylosing spondylitis and HLA-B27 (15). Considering the fact that anti-LGI1 encephalitis is a disease caused by antibodies, it can be inferred that the disease is more closely associated with HLAII genes.

HLA class IIplays an important role in other humoral autoimmunity as well. Type 1 Diabetes (T1D) is characterized by destruction of islet β-cells. Insulin autoantibodies (IAA) as corresponding autoantibodies appear in children with DR4–DQ8 haplotype, which is located on HLA class II and can influence both etiology and pathogenesis of T1D (16). The HLA complex accounted for about 50% of genetic risk of T1D (17), and the risk of progression is conferred by specific HLA-DR/DQ alleles, while some haplotypes (i.e., DR2) could be protective factors (18). T lymphocyte differentiation, characterized by HLA, is identified as an independent pathway involved in systemic lupus erythematosus (SLE) susceptibility. Genes in the HLA region dominated associated genes in the T cell differentiation and antigen processing and presentation pathways, which was confirmed by gene-based association testing (19).

To date, HLA genotyping as well as genome-wide association study (GWAS) have been performed among patients with anti-LGI1 encephalitis across multiple populations, including Caucasian population (7–10), South Korean population (6), and southwestern Han Chinese population (11), demonstrating a significant association between unique HLA subtypes and anti-LGI1 encephalitis. In parallel, the frequencies of some definite sites located on major histocompatibility complex (MHC) class II were significantly higher in the anti-LGI1 encephalitis group than in the healthy control or epilepsy groups. Therefore, it is speculated that HLA isotypes might activate the immune response or work through initiating T–B cell interactions during disease onset (8). However, it should be noted that HLA subtypes in published studies are not consistent, possibly due to different ethnicities.

The first research on genetic susceptibility for anti-LGI1 encephalitis was conducted in the Netherlands (7). Interestingly, researchers explored the relationship between HLA and anti-LGI1 patients with or without tumors. They found a strong correlation of non-tumor anti-LGI1 encephalitis with HLA-DR7 and HLA-DRB4, as significant as the correlation between HLA-B27 and patients with ankylosing spondylitis. It suggested that the deficiency of HLA-DR7 or DRB4 appeared to boost the prevalence of a tumor. Consequently, the researchers recommended an intensive tumor screen and long-term follow-up in anti-LGI1 encephalitis without HLA-DR7 or DRB4.

Conversely, a British research (8) revealed the uncorrelation between HLA and tumor in anti-LGI1 encephalitis patients. Hence, more studies in HLA and tumor in these patients are urgent to guide the clinical diagnosis and treatment. Moreover, this study found that the HLA class I and IIvariants (HLA-DRB1^*^07:01, HLA-DQA1^*^02:01, HLA-B^*^57:01) may increase the risk of adverse drug reactions (20).

The first study implementing genome-wide association (GWAS) analysis was conducted in Germany (9). The unprecedented discovery was that anti-LGI1 encephalitis was highly associated with 27 single-nucleotide polymorphisms (SNPs) located in the HLA class II region between HLA-DRB1 and HLA-DQA1 (leading SNP rs2858870) in the region of MHCIIgenes. It even found that DRB1^*^07:01 and DQA1^*^02:01 always appeared together in all participants. Considering that related alleles were associated with decreased total serum IgG levels, and LGI1 autoantibodies mainly belong to the IgG4 subclass (21), the result suggested that these haplotypes, in addition to improving peptide presentation of LGI1 peptides, may be associated with the disorder of the IgG4-LGI1, which is potentially pathogenic. In addition, a study in South Korea (6) discovered a higher frequency of B^*^44:03 and C^*^07:06 alleles of the HLA class I in anti-LGI1 encephalitis patients, illustrating that HLA class I is possible pathogenesis of anti-LGI1 encephalitis as well.

HLA genotyping was performed in anti-LGI1 encephalitis cohort in France recently (10) and found that 88% of patients carried DRB1^*^07:01. The study newly identified that non-carriers were younger, more frequently women, and presented less frequently with psychiatric and frontal symptoms, whereas non-carriers were not associated with poor outcomes. The HLA association in paraneoplastic or oncological patients has not been confirmed. The mechanisms of sex and age bias in HLA class II-associated diseases are unclear, while a study presumed that estrogens may change HLA expression (22).

The only study in the Chinese Han population was conducted in China (11), however, the study showed no evidence that the DRB1^*^07:01 ~ DQB1^*^02:02 haplotype was associated with this disease. Researchers attributed this inconsistency to ethnic differences. All of the results of these researches mentioned above possessed homogeneity and heterogeneity, seen in Table S1.

WES is a promising tool in genetic testing methods, which offers the possibility of identifying rare or novel alleles responsible for the disease. In 2014, a child with cerebral lupus was identified a homozygous mutation in the Three Prime Repair Exonuclease 1 (TREX1) by this method (23). Some studies found possible mechanisms as TREX1^R97H^ mutant protein had a severe reduction in exonuclease activity that leads to defects in clearance of nucleic acids, and triggers signaling pathways that promote secretion of type I IFNs and inflammation.

Herein, we performed HLA typing and WES on Patient 1. His HLA typing was DRB1^*^07:01, DQA1^*^02:01, and DQB1^*^03:03 (Table 2), which perfectly matched the H3 haplotype reported by the previous study from Germany. HLA-B^*^57:01 was also detected, which has been shown to perhaps induce adverse drug reactions (20). It should be noted that these alleles were recurrent in studies based on multiple populations, which indicated a highly possible association with the clinical onset of anti-LGI1 encephalitis. Given that this disease mainly occurs in middle-aged and elderly individuals, the pathogenesis may also be associated with environmental effects. According to the result of WES, we found one homozygous HLA-DRB1 variant (NM_002124:exon2:c.101-1G>A) and one heterozygous HLA-DPA1 variant (NM_001242524:exon5:c.746G>A:p.R249H), and details are given in Table S2. These variants were absent or rare in the general Chinese population. Regrettably, these mutations have not been verified in his sister and pedigree, and could not be clarified the exact role in the disease process. Apart from HLA variants, investigation on WES did not reveal any disease-causing variants associated with anti-neuronal autoimmune encephalitis. This unremarkable result was possibly associated with undetected problems in gene expression or epigenetics. In addition, tumor screening of two patients was performed by serum tumor markers and chest CT, not in the whole body, which was a limitation. Hence, there is an urgent need to verify these speculations by expanding the samples and performing further genome-wide association analysis.

**Table 2:** Result of Patient 2's HLA genotyping.

**Allels/haplotypes**	**HLA-A**	**HLA-B**	**HLA-C**	**HLA-DRB1**	**HLA-DQB1**	**HLA-DQA1**	**HLA-DRB4**	**HLA-DPB1**
	02:03,33:03	38:02,57:01^*^	06:02,07:02	07:01^*^,13:12	03:01,03:03^*^	02:01^*^,05:03	01:03^*^,/	05:01,13:01

* *Special HLA genotypes that appeared in the literature studies on genetic susceptibility for anti-LGI1 encephalitis*.

In summary, the latest studies above have confirmed that anti-LGI1 encephalitis is genetically susceptible, highly associated with specific alleles located on HLA class II. However, more researches with large samples and more races are necessary to verify it. In addition, the frequencies of these HLA variants were much higher than the prevalence of anti-LGI1 encephalitis, suggesting that people with unique alleles may not develop the disease. Therefore, further studies should focus on the possibility of other influencing factors of anti-LGI1 encephalitis, for instance, additional haplotypes, environmental impacts, and other random effects.

## Data Availability Statement

The original contributions presented in the study are included in the article/Supplementary Material, further inquiries can be directed to the corresponding author.

## Ethics Statement

The studies involving human participants were reviewed and approved by the First Affiliated Hospital of Anhui Medical University. The patients/participants provided their written informed consent to participate in this study. Written informed consent was obtained from the individual(s) for the publication of any potentially identifiable images or data included in this article.

## Author Contributions

CD explained the data and wrote the manuscript. QS, RL, and HL acquired and analyzed data. CD and YW revised the manuscript. All authors approved the final manuscript.

## Funding

This study was financially supported by the National Natural Science Foundation of China (YW, Grant No. 82071460).

## Conflict of Interest

The authors declare that the research was conducted in the absence of any commercial or financial relationships that could be construed as a potential conflict of interest.

## Publisher's Note

All claims expressed in this article are solely those of the authors and do not necessarily represent those of their affiliated organizations, or those of the publisher, the editors and the reviewers. Any product that may be evaluated in this article, or claim that may be made by its manufacturer, is not guaranteed or endorsed by the publisher.
