# Supplementary material for: The First Case of Familiar Anti-leucine-rich Glioma-Inactivated1 Autoimmune Encephalitis: A Case Report and Literature Review
Source: Front Neurol. 2022 Apr 14;13:855383. doi: 10.3389/fneur.2022.855383 (PMC9047818; doi:10.3389/fneur.2022.855383)
Supplement: Supplementary file 1 [file Data_Sheet_1.docx]

Supplementary Material

# Supplementary Tables

**Supplementary Table 1:** Comparison of existing studies on the genetic susceptibility between HLA and anti- LGI1 encephalitis.

| Ethnic groups | Cases | Healthy controls | HLA class Ⅱ allels or haplotype | HLA class Ⅰ allels or haplotype | New findings |
| --- | --- | --- | --- | --- | --- |
| Caucasian descent (Dutch) | 29 | 5604 | HLA-DRB4, HLA-DR7 | - | Absence of HLA-DR7 or DRB4 may increase the probability of a tumor |
| Asian population (Korean) | 11 | 485 | HLA-DRB1*07:01-DQB1*02:02 haplotype | HLA-B*44:03 and C*07:06 alleles | - |
| Caucasian descent (British) | 68 | 5553 | DRB1*07:01-DQA1*02:01-DQB1*02:02 haplotype / DRB1*07:01-DQA1*02:01-DQB1*03:03 / DPA1*02:01-DPB1*11:01 | HLA-C*06:02-B*57:01 haplotype | HLADRB1*07:01, HLA-DQA1*02:01, HLA-B*57:01) may increase risk of adverse drug reactions / no significant HLA differences between tumor and non-tumor cases |
| Caucasian descent (German) | 75 | 1194 | DRB1*07:01 and DQA1*02:01 haplotype / DRB1*07:01, DQA1*02:01 and DQB1*02:02 haplotype / DRB1*07:01, DQA1*02:01, and DQB1*03:03 haplotype | - | - |
| Southwestern Han Chinese descent (Chinese) | 11 | 200 | DRB1*03:01 or DQB1*02:01 alleles and DRB1*03:01-DQB1*02:01 | - | - |
| Most Caucasian descent (French) | 72 | 300 | DRB1*07:01 / DQB1*02:02 / DRB1*04:02 | HLA-A*30:01 and C*06:02 allelss | Absence of HLA-DR7 may decrease the frequency of psychiatric symptoms and frontal syndrome |

**Supplementary Table 2:** HLA-DPA1 and DRB1 variants in Patient 1.

|  | HLA-DPA1 | HLA-DRB1 |
| --- | --- | --- |
| cDNA mutation | c.746G>A | c.101-1G>A |
| Protein alteration | p.R249H | - |
| Variant type | Heterozygous | Homozygous |
| 1000G_eas | - | - |
| gnomAD_exome_eas | 0 | 0 |
| gnomAD_gnome_eas | 0 | 0 |
| SIFT | T | - |
| PolyPhen-2 | B | - |
| MutationTaster | N | D |
| CADD Phred | 10.87 | 33 |

Abbreviations: 1000G_eas, East Asian population of 1000 Genomes Project; gnomAD, the Genome Aggregation Database; -, not available; *T*, tolerated; *B*, benign; *N*, polymorphism; *D*, disease causing

# Supplementary Figures


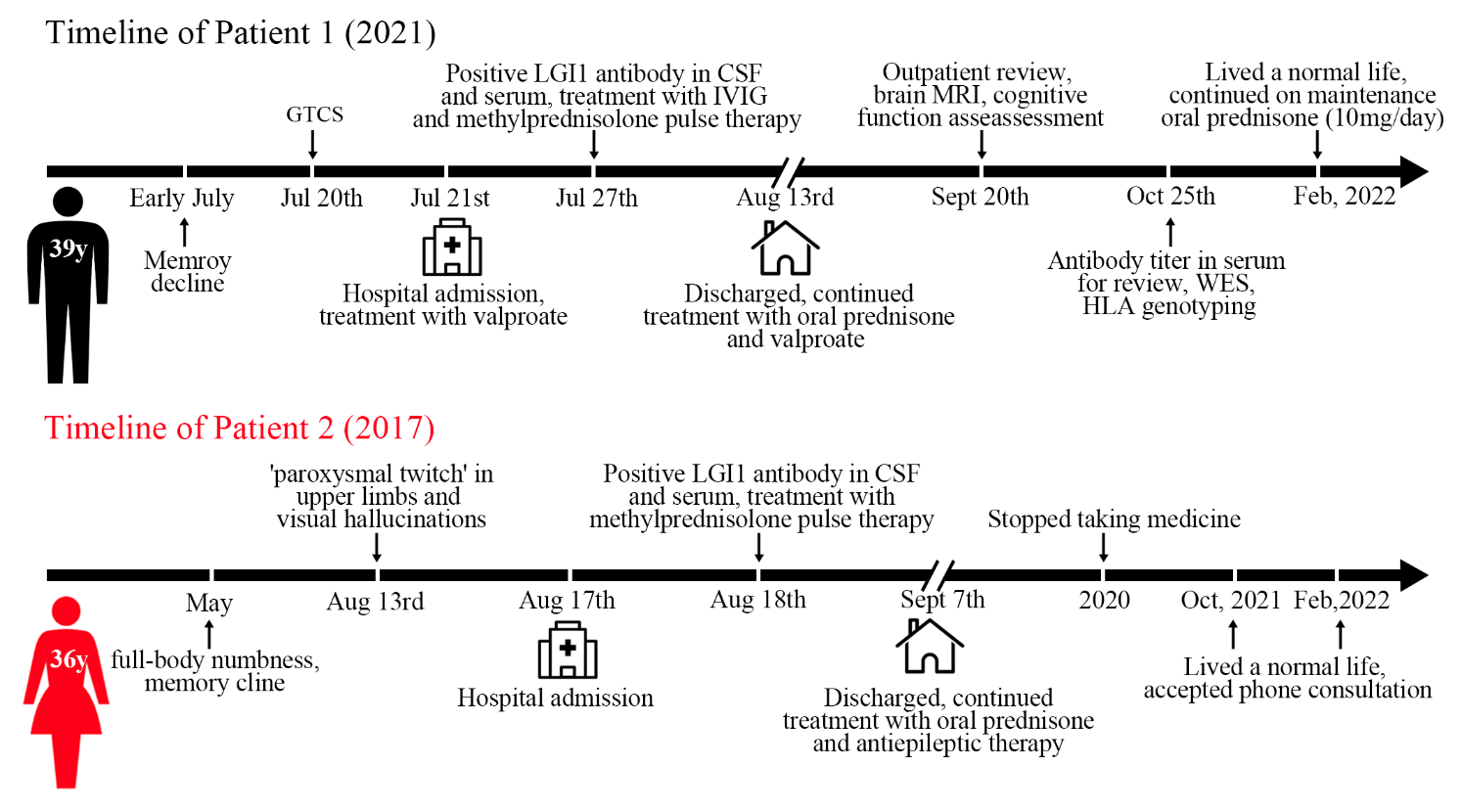
**Supplementary Figure 1:**  Timeline of two patients


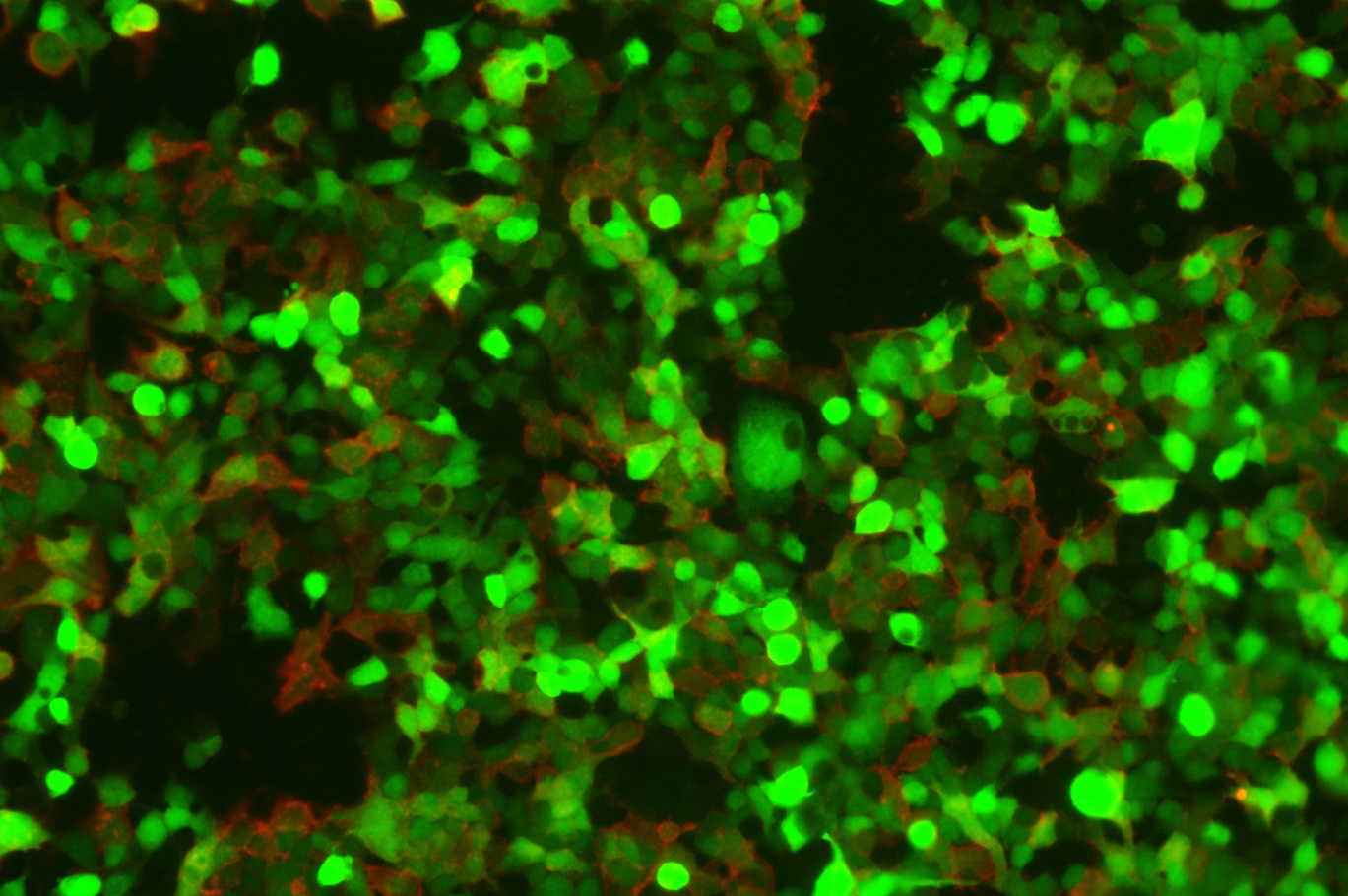


**
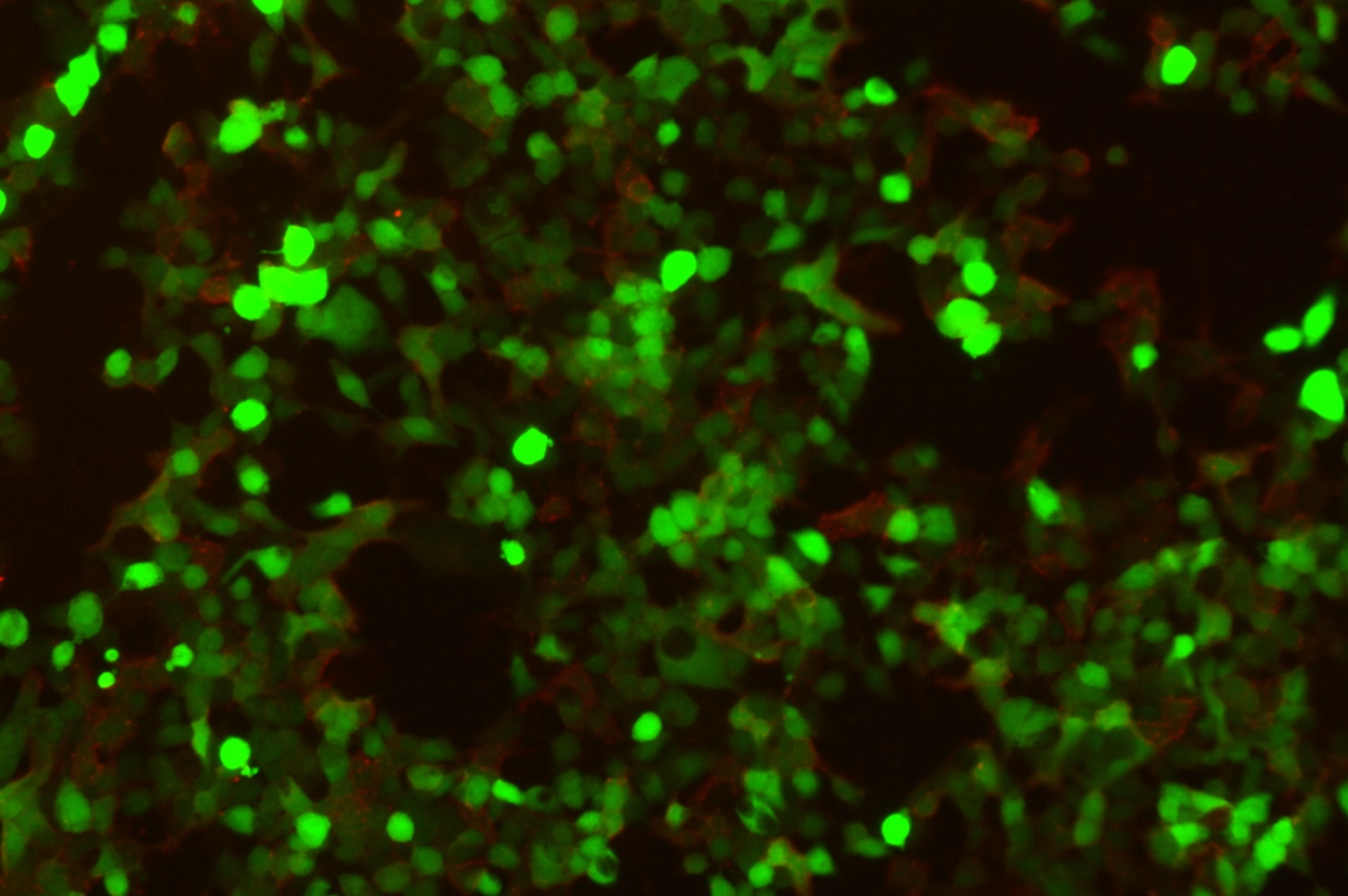
Supplementary Figure 2 (A-B)**. Immunofluorescence assay graphs. Before Patient 1 received treatment, the autoimmune encephalitis antibody test was performed and showed positive LGI1 antibody in serum (The titer was 1:1000+) (Image shown in supplementary figure 2-A). After 3 months of treatment, the autoimmune encephalitis antibody test in serum showed that anti-LGI1 titer was lowered to 1:100+ (Image shown in supplementary figure 2-B)

**Supplementary methods:**

**1. Determination of autoimmune antibodies**

Levels of LGI1 antibody in serum and CSF of two patients were performed as soon as they were admitted to hospital, and measured by cell-based immunofluorescence assay (CBA), also included other antibodies, such as NMDA, GABA, AMPAR1, AMPAR2, CASPR2, DPPX, GAD65 antiobodies, et al.

**2. HLA genotyping**

HLA typing was performed by the Huada Company (HuaDa, Shenzhen, China). Genomic DNA from the blood sample of Patient 1 was extracted using a MagPure Buffy Coat DNA Midi KF Kit (Magen, China). High-resolution sequence-based HLA typing with Sanger sequencing-based typing (SBT) was performed on a DNA sequencer (Applied Biosystems 3730XL, USA). HLA-A, B, C, DRB1, DQB1, D1A1, DRB4, and DPB1 were determined at the four-digit allele level. Exon 2,3 and 4 were sequenced for DPB1, and exon 2 and 3 were sequenced for DQA1 and DRB4.

**3. Whole Exome Sequencing (WES) and massively parallel sequencing data processing**

WES was performed by Genesky Biotechnologies Inc (Shanghai, China). The exomes were captured by Agilent SureSelect Human All Exon Kit (Agilent Technologies, CA, USA), and high-throughput sequencing was performed using the Illumina HiSeq (Illumina, CA, USA). Evaluation of DNA Quality, bioinformatics analysis, and sequence alignment was completed by Genesky Biotechnologies Inc.

The sequencing reads were aligned to human genome (*hg38*) using the Burrows–Wheeler Aligner (BWA). Before variant calling, sequence alignment files were generated to duplicate removal, local realignment around known indels and base quality recalibration using the Genome Analysis Toolkit (*GATK*). Variations that included single-nucleotide variants (SNVs) and small insertions or deletions (indels) were identified using *GATK* with variant quality score recalibration (VQSR) protocol, and further filtered using a recommended threshold value (mapping quality > 30, base quality >20, and read numbers > 3). Then SNVs was annotaiton by *ANNOVAR*. To sort potentially deleterious variants from benign polymorphisms, *Perl* scripts were used to filter the SNVs against those of   SNV recorded in dbSNP150 and with a minor allele frequency of ≥1% in Chinese from 1000 genome database was considered as benign polymorphisms and therefore removed for subsequent analysis.
